# Increased Lipoprotein-associated phospholipase A2 activity portends an increased risk of resistant hypertension

**DOI:** 10.1186/s12944-016-0184-9

**Published:** 2016-01-22

**Authors:** Zhiming Li, Jingguang Liu, Yuansheng Shen, Fanfang Zeng, Dongdan Zheng

**Affiliations:** Department of Cardiology, Huizhou Municipal Central Hospital, 41st Eling North RD, Huicheng District, Huizhou, China; Department of Cardiology, Shenzhen Sun Yat-sen Cardiovascular Hospital, Shenzhen, China; Department of Cardiology, The First Affiliated Hospital of Sun Yat-sen University, Guangzhou, China

**Keywords:** Lipoprotein-associated phospholipase A2, Resistant hypertension, Relationship

## Abstract

**Background:**

To investigate the relationship between plasma lipoprotein-associated phospholipase A2 (Lp-PLA2) activity and incidence of resistant hypertension (RH).

**Methods:**

This was a cross-sectional research. In essential, it was an observational design and collecting data on a population at a single point in time to evaluate the associations of studied variables. Totally 208 patients with arterial hypertension were enrolled. Baseline characteristics were collected and fasting venous blood were drawn for plasma Lp-PLA2 activity assessment. Twenty-four hour ambulatory blood pressure ambulatory (ABPM) was performed to diagnose RH. Initially, based on ABPM examination, all participants were divided into two groups, namely RH group and without RH group. And thereafter, in order to evaluate the effects of Lp-PLA2 activity on blood pressure, all participants were divided into low (< 225 nm/min/ml) and high (≥ 225 nm/min/ml) Lp-PLA2 activity groups based on the cut-off value of Lp-PLA2 activity. Comparisons were conducted between groups.

**Results:**

Forty two patients were diagnosed as RH. Compared to patients without RH, patients with RH were more elderly, had more males, smokers, longer duration of hypertension, higher plasma C-reactive protein (CRP) level and Lp-PLA2 activity (*P* < 0.05 for all comparisons). More RH patients treated with calcium channel blocker and diuretic, while less treated with angiotensin converting enzyme inhibitor, angiotensin receptor blocker and statins (*P* < 0.05 for all comparisons). Compared to low Lp-PLA2 group, the rate of RH was significantly higher in high Lp-PLA2 group (26.7 % versus 6.1 %, *P* < 0.05). Multivariate regression analysis revealed that after adjusted for age, gender, smoking, body mass index, hypertension duration, CRP, and anti-hypertensive drugs, association between Lp-PLA2 activity and RH remained significant, with odds ratio (OD) of 2.02 (95 % confidence interval, CI 1.85-2.06, *P* < 0.05). Nonetheless, the association was attenuated when further adjusted for statins, with OR of 1.81 (95 % CI 1.74-1.93, *P* < 0.05).

**Conclusion:**

Increased plasma Lp-PLA2 activity portends increased risk of RH, and statins may be beneficial to reduce incidence of RH in subjects with increased plasma Lp-PLA2 activity.

## Background

Arterial hypertension is a major modifiable risk factor of cardiovascular diseases around the world [1, 2]. And resistant hypertension (HR), which is defined as blood pressure remains above 140/90 mm Hg despite treatment on 3 different classes of anti-hypertensive drugs (including one diuretic) at their optimal doses [3], significantly increases the risk of cardiovascular events such as heart failure, renal dysfunction and ischemic stroke [4]. It was previously reported that in patients with resistant hypertension, plasma level of inflammatory cytokine such as C-reactive protein (CRP) was considerably elevated [5, 6], and the underlying mechanisms operating in these processes are not fully investigated yet.

Lipoprotein-associated phospholipase A2 (Lp-PLA2) is a serine-dependent lipase which circulates in blood stream after released from inflammatory cells within vascular atherosclerotic plaques [7]. Compared to CRP, Lp-PLA2 is a more specific biomarker of vascular inflammation [8]. A substantial amount of studies reveal that vascular inflammation contributes to endothelial dysfunction, which is reflected as decreased nitric oxide (NO) generation and increased endothelin-1 (ET-1) production [9, 10]. The imbalance between NO and ET-1 generation may lead to peripheral resistant vessels constriction and blood pressure elevation. With respect to the potent effects of Lp-PLA2 on promoting vascular inflammation, we therefore hypothesized that increased plasma Lp-PLA2 activity might be associated with the incidence of resistant hypertension. In our present cross-sectional research, we compared plasma activity Lp-PLA2 between subjects with and without RH. The major objective of present research was to investigate whether increased Lp-PLA2 activity was significantly and independently associated with the incidence of resistant hypertension. We considered that the results from our present research might shed insights for further studies in investigating an effective therapy for managing resistant hypertension.

## Methods

### Participants’ enrollment and studied protocol

In brief, in outpatient department, subjects previously diagnosed as primary arterial hypertension or reported taking anti-hypertensive drugs were enrolled after oral informed consent was obtained. All recruited subjects were clearly informed about the design of present study, in terms of only laboratory examination and 24 h ambulatory blood pressure monitoring (ABPM) would be conducted and no intervention including blood pressure managing would be conducted. Those with heart failure, liver or renal dysfunction, cognitive impairment, documented cancer and connective tissue disease, secondary hypertension would be ruled out. Totally 208 patients with primary arterial hypertension were recruited. Demographics and clinical characteristics were collected by questionnaire. Present medications used such as anti-hypertensive drugs, anti-platelet and statins were recorded. Briefly, patients without RH had higher percentage of statins treatment than those with RH. Fasting venous blood were drawn for laboratory examination. In brief, plasma Lp-PLA2 activity was measured by using an automated Colorimetric Activity Method assay (diaDexus Inc., South San Francisco, CA) using a Beckman Coulter (Olympus) AU400e autoanalyzer. All procedures were conducted in accordance to the manual’s instruction and the inter-assay variation coefficient of Lp-PLA2 activity assay was 4.0 %. Briefly, this is a cross-sectional research. By definition, cross-sectional research is an observational design, and collecting data on a population at a single point in time to evaluate the associations of studied variables.

### Diagnosis of resistant hypertension

In order to reduce the incidence of “white-coat” hypertension or masked hypertension, 24 h ABPM was performed on top of official blood pressure examination. On the basis of 24 h ABPM readings, patients with mean 24 h systolic blood pressure (SBP) > 130 mm Hg or diastolic blood pressure (DBP) > 80 mm Hg were diagnosed as resistant hypertension, in spite of treatment on 3 different classes of anti-hypertensive drugs at their optimal doses (including one diuretic) [3]. And no change of anti-hypertensive drugs would be conducted after enrolment.

### Studied groups

Initially, based on 24 h ABPM examination, all participants were divided into two groups, namely resistant hypertension group and without resistant hypertension group. And thereafter, in order to evaluate the effects of Lp-PLA2 activity on blood pressure, all participants were divided into low (< 225 nm/min/ml) and high (≥ 225 nm/min/ml) Lp-PLA2 activity groups based on the cut-off value of Lp-PLA2 activity [11].

### Statistical analyses

Continuous data was presented as mean ± SD and was compared by the Student’s *t*-test when data was normally distributed, otherwise was compared by the Wilcoxon rank-sum test. Categorical data was presented as percentage and was compared by *χ*^2^ test. The relationship between plasma Lp-PLA2 activity and resistant hypertension was using multivariate regression analyses. All reported *p* values were 2-sided, and a *p* value of < 0.05 was considered statistically significant. All statistical analyses were conducted with the SPSS statistical package for Windows version 19.0 (SPSS Inc., Chicago, Illinois).

## Results

### Baseline characteristics of all participants

Baseline characteristics of all participants were presented in Table 1. The percentage of RH in our present cross-sectional research was nearly 20.2 %. Generally, as compared to patients without RH, patients with RH were more elderly (59.3 ± 10.1 years versus 53.2 ± 11.4 years, *P* = 0.018), had higher percentages of male (76.2 % versus 62.7 %, *P* < 0.001) and smokers (64.3 % versus 60.2 %, *P* = 0.037), longer duration of hypertension (6.5 ± 3.7 years versus 4.0 ± 2.1 years, *P* = 0.022), higher CRP level (24.7 ± 5.0 mg/L versus 15.2 ± 5.4 mg/L, *P* < 0.001) and Lp-PLA2 activity (254.2 ± 26.7 nmol/min/mL versus 228.1 ± 24.5 nmol/min/mL, *P* < 0.001). Briefly, most of these variables have had been previously demonstrated as significant risk factors of resistant hypertension. Expectedly, compared to those without RH, office SBP (147.3 ± 3.6 mm Hg versus 132.4 ± 5.5 mm Hg, *P* < 0.001) and DBP (94.4 ± 2.6 mm Hg versus 82.3 ± 4.4 mm Hg, *P* < 0.001), and 24 h mean SBP (137.7 ± 6.2 mm Hg versus 126.3 ± 4.9 mm Hg, *P* < 0.001) and DBP (86.5 ± 4.7 mm Hg versus 76.8 ± 2.4 mm Hg, *P* < 0.001) were all significantly higher in patients with RH. With respect to the usage of anti-hypertensive medicines, more patients with RH treatment on calcium channel blocker and diuretic, and less treatment on angiotensin converting enzyme inhibitor and angiotensin receptor blocker. Patients without RH had higher percentage of statins treatment than those with RH (38.1 % versus 53.6 %, *P* < 0.001).

**Table 1:** Baseline characteristics of all participants

Variables	Overall (*n* = 208)	Without RH (*n* = 166)	With RH (*n* = 42)	*P* for trend
Age (years)	55.7 ± 12.3	53.2 ± 11.4	59.3 ± 10.1	0.018
Male, *n* (%)	136 (65.4)	104 (62.7)	32 (76.2)	< 0.001
Smoking, *n* (%)	127 (61.1)	100 (60.2)	27 (64.3)	0.037
BMI (Kg/m^2^)	24.4 ± 3.2	23.3 ± 2.8	24.9 ± 3.0	0.87
Duration of hypertension (years)	5.4 ± 3.3	4.0 ± 2.1	6.5 ± 3.7	0.022
Diabetes, *n* (%)	38 (18.3)	30 (18.1)	8 (19.0)	0.51
CRP (mg/L)	19.3 ± 6.5	15.2 ± 5.4	24.7 ± 5.0	< 0.001
TC (mmol/L)	5.2 ± 1.1	5.1 ± 1.0	5.3 ± 1.1	0.63
LDL-C (mmol/L)	3.6 ± 0.8	3.5 ± 0.9	3.6 ± 0.7	0.66
HDL-C (mmol/L)	1.1 ± 0.3	1.0 ± 0.2	1.1 ± 0.5	0.57
TG (mmol/L)	1.8 ± 0.4	1.8 ± 0.5	1.7 ± 0.3	0.49
Creatinine (μmol/l)	116.9 ± 12.74	110.2 ± 10.56	120.7 ± 9.86	0.28
BUN (mmol/L)	6.6 ± 2.4	6.7 ± 2.0	6.3 ± 2.2	0.43
Lp-PLA2 activity (nmol/min/mL)	230.5 ± 26.4	228.1 ± 24.5	254.2 ± 26.7	< 0.001
Official SBP (mm Hg)	140.7 ± 10.4	132.4 ± 5.5	147.3 ± 3.6	< 0.001
Official DBP (mm Hg)	87.6 ± 5.9	82.3 ± 4.4	94.4 ± 2.6	< 0.001
24 h mean SBP (mm Hg)	134.5 ± 5.6	126.3 ± 4.9	137.7 ± 6.2	< 0.001
24 h mean DBP (mm Hg)	83.3 ± 4.8	76.8 ± 2.4	86.5 ± 4.7	< 0.001
Heart rate (beats per minute)	81.4 ± 11.5	78.7 ± 10.3	88.2 ± 9.0	< 0.001
Number of anti-hypertensive medicines	3.4 ± 0.5	3.3 ± 0.4	3.5 ± 0.4	0.65
ACEI/ARB, *n* (%)	167 (80.3)	135 (81.3)	32 (76.2)	0.044
CCB, *n* (%)	115 (55.3)	89 (53.6)	26 (61.9)	0.037
Beta-blocker, *n* (%)	87 (41.8)	69 (41.2)	18 (42.9)	0.71
Diuretic, *n* (%)	186 (89.4)	144 (86.7)	42 (100)	< 0.001
Alpha-blocker, *n* (%)	45 (21.6)	36 (21.7)	9 (21.4)	0.83
Statins, *n* (%)	105 (50.5)	89 (53.6)	16 (38.1)	< 0.001
Aspirin, *n* (%)	167 (80.3)	135 (81.3)	32 (76.2)	0.058

Denote: *RH* resistant hypertension, *BMI* body mass index, *TC* total cholesterol, *LDL-C* low density lipoprotein cholesterol, *HDL-C* high density lipoprotein cholesterol, *TG* triglyceride, *BUN* blood urine nitrogen, *ACEI* angiotensin converting enzyme inhibitor, *ARB* angiotensin receptor blocker, *CCB* calcium channel blocker, Diuretic includes thiazide, loop and aldosterone antagonist

### Blood pressure comparisons between low and high Lp-PLA2 activity groups

Blood pressure comparisons between low and high Lp-PLA2 groups were conducted. As shown in Table 2, patients with high Lp-PLA2 activity (≥ 225 nm/min/ml) had significantly higher office SBP and DBP, and 24 h mean SBP and DBP (*P* < 0.05 for all comparisons) as compared to low Lp-PLA2 activity group (< 225 nm/min/ml). Moreover, the percentage of RH (26.7 % versus 6.1 %, *P* < 0.05) was also significantly higher in patients with high Lp-PLA2 activity than those with low Lp-PLA 2 activity.

**Table 2:** Lp-PLA2 activity and blood pressure

Variables	< 225 nm/min/ml	≥ 225 nm/min/ml	*P* value
Number (%)	66 (31.7)	142 (68.3)	< 0.001
Official SBP (mm Hg)	135.4 ± 13.7	148.7 ± 9.4	< 0.001
Official DBP (mm Hg)	85.3 ± 6.3	93.5 ± 5.5	< 0.001
24 h mean SBP (mm Hg)	127.8 ± 9.2	138.4 ± 10.5	< 0.001
24 h mean DBP (mm Hg)	76.5 ± 6.3	86.5 ± 5.7	< 0.001
RH, n (%)	4 (6.1)	38 (26.7)	< 0.001

### Relationship of Lp-PLA2 activity and incidence of resistant hypertension

Relationship of Lp-PLA2 activity and incidence of RH was evaluated by multivariate regression analysis. In the model 1, after adjusted for age and gender, odds ratio (OR) for RH was 2.04 (95 % confidence interval, CI 1.87–2.08, *P* < 0.05) in the high Lp-PLA2 activity group versus the low Lp-PLA2 activity group. And the strength of this association remained similar after further adjusted for body mass index (BMI), smoking, hypertension duration, CRP, TC, LDL-C and anti-hypertensive drugs (model 2), with OR of 2.02 (95 % CI 1.85–2.06, *P* < 0.05). Nonetheless, the association was attenuated when further adjusted for statins therapy (model 3), with OR of 1.81 (95 % CI 1.74-1.93, *P* < 0.05).

## Discussion

Our present cross-sectional research reveals that in patients with primary arterial hypertension, the rate of RH is nearly 20 % as diagnosed by 24 h ABPM. As compared to patients without RH, those with RH have more co-morbidities such as more elderly, larger percentages of male patients and smokers, longer duration of hypertension and have higher plasma CRP level. The novel finding of our present research is that plasma Lp-PLA2 activity in patients with RH is significantly higher and increased Lp-PLA2 activity is independently associated with the incidence of RH. Statins therapy may be helpful to reduce the incidence of RH in subjects with increased plasma Lp-PLA2 activity.

As is well known that arterial hypertension contributes to a variety of CVD, and effectively lowering blood pressure below 140/90 mm Hg could significantly reduce the incidence and prevalence of heart failure, renal dysfunction and ischemic stroke. Accordingly, the prevalence of RH is between 10–20 % and the health burden attributable to RH is substantial [3]. Therefore, it is clinically important to decrease the incidence and prevalence of RH. Previously, a substantial amount of studies revealed that poor adherence, “white-coat” effect and unrecognized co-morbidities such as obstructive sleep apnea and primary hyper-aldosteronism were the underlying mechanisms associated with pseudo-resistant hypertension [12–14]. In addition, it was reported that plasma level of inflammatory cytokine such as CRP in patients with RH was also increased [6]. However, whether increased plasma CRP level was independently associated with RH was unclear yet.

Compared to CRP (an unspecific inflammatory biomarker), Lp-PLA2 is a highly specific marker of vascular inflammation. It has been demonstrated that the higher the plasma Lp-PLA2 activity, the more severe of vascular inflammation and endothelial dysfunction [15, 16]. Endothelial dysfunction is associated with peripheral resistant vessels constriction and blood pressure elevation. Therefore, we considered that increased Lp-PLA2 activity might be associated with the incidence of RH. And data from our present study revealed that as compared to patients without RH, Lp-PLA2 activity in patients with RH was significantly higher (254.2 ± 26.7 nmol/min/mL versus 228.1 ± 24.5 nmol/min/mL, *P* < 0.001). Moreover, on the basis of the U.S Food and Drug Administration recommendation (which defines Lp-PLA2 activity of more than ≥ 225 nm/min/ml is beyond normal range) [11], all participants were divided into two groups as indicated in Table 2. Comparisons of blood pressure revealed that compared to those with low Lp-PLA2 activity, patients with high Lp-PLA2 activity had higher office SBP and DBP, and 24 h mean SBP and DBP, and the incidence of RH was also significantly higher in high Lp-PLA2 activity group (26.7 % versus 6.1 %, *P* < 0.001). We considered that the following aspects might partially explain our findings. In the first place, as mentioned above that vascular inflammation and endothelial dysfunction induced by Lp-PLA2 played a critical role on blood pressure elevation. Secondly, sympathetic nerve activation triggered by chronic systemic inflammation might also lead to catecholamine release and renin-angiotensin axis activation. Both of these pathophysiological changes could cause blood pressure elevation through peripheral resistant vessels constriction and fluid retaining [17]. Although we haven’t measured plasma level of catecholamine, heart rate in patient with RH was significantly higher than those without RH might indirectly reflect the activation of sympathetic nerve system. Last but not the least, it was reported that in patients with coronary artery disease, Lp-PLA2 was associated with arterial stiffness [18]. Therefore, we postulated that through enhancing arterial stiffness, Lp-PLA2 might directly increase blood pressure.

Finally, multivariate regression analysis revealed that after extensively adjusted for potential confounding factors including age, smoking, duration of hypertension, CRP, BMI, and anti-hypertensive medicines, Lp-PLA2 activity was still significantly associated with RH. Nonetheless, the relationship was attenuated by statins, and the underlying mechanism we considered might be due to improvement of vascular inflammation and endothelium function by statins treatment, which was beneficial for vessel dilation and blood pressure reduction [19].

There were some strengths and weaknesses of our present research. To our best knowledge, this was the first study to evaluate the relationship between Lp-PLA2 activity and the incidence of RH. In addition, 24 h ABPM was used to diagnose RH which could avoid the “white-coat” hypertension or masked hypertension. Nonetheless, since our present research was a cross-sectional research, therefore, the causal relationship between Lp-PLA2 activity and the incidence of RH could not be drawn. Moreover, despite we extensively adjusted for confounding factors, inherent biases of cross-sectional research still could not be avoided.

## Conclusion

Data from our present study indicate that compared to those without RH, plasma Lp-PLA2 activity in patients with RH is significantly higher, suggesting that increased plasma Lp-PLA2 activity is associated with the incidence of RH. Lp-PLA2 activity may be used as a marker to identify those who are at increased risk of RH. Future studies are warranted to investigate whether Lp-PLA2 inhibitor or statins could be used to reduce the incidence of RH.
